# Supplementary material for: WordCommentsAnalyzer: A windows software tool for qualitative research
Source: F1000Res. 2018 Sep 4;7:536. Originally published 2018 May 3. [Version 2] doi: 10.12688/f1000research.14819.2 (PMC6137411; doi:10.12688/f1000research.14819.2)
Supplement: Supplementary file 3 [file f1000research-7-17614-s0002.tgz › b2a79496-4ada-47c4-9682-a99ce867836d.zip!/Eight Iranian Health Journals/Archives of Rehabilitation_5.docx]

© Archives of Rehabilitation (RJ). University of Social Welfare and Rehabilitation Sciences, Koodakyar Alley, Daneshjoo Blv., Evin, Tehran, Iran. This work is licensed under [CC BY-NC 4.0](https://creativecommons.org/licenses/by-nc/4.0/).

Volume & Issue: دوره 18 - شماره 1

Title: مقایسه تغییر توجه و خلاقیت در کودکان دوزبانه و تک‌زبانه

http://rehabilitationj.uswr.ac.ir/article-1-1911-fa.html

هدف برخی از تحقیقات گزارش داده‌اند که دوزبانگی ممکن است فرایندهای شناختی را تحت تأثیر قرار دهد. فراگیری زبان دوم از طریق مؤسسات آموزش زبان و با استفاده از کلاس‌های آموزشی تشکیل‌شده در این مؤسسات صورت می‌گیرد. این کلاس‌ها در عصر حاضر به‌سرعت در حال گسترش است. با توجّه به ماهیت متمایز فراگیری زبان‌های خارجی از طریق مؤسسات آموزشی نسبت به روش‌های دیگر دوزبانه‌شدن، اثرات فراگیری زبان خارجی از طریق آموزشگاه‌های زبان سزاوار خط جداگانه‌ای در تحقیقات دوزبانگی است. لذا هدف پژوهش حاضر مقایسه سوئیچینگ و خلاقیت در کودکان زبان‌آموز سطوح پیشرفته زبان انگلیسی و کودکان غیر زبان‌آموز است. روش بررسی روش پژوهش از نوع علّی‌مقایسه‌ای است. پژوهش حاضر به روش مقطعی انجام شده است. جامعه مطالعه‌شده در این پژوهش شامل تمام کودکان (13 تا 15 ساله) تک‌زبانه و دوزبانه شهر اصفهان در سال 1394 است. به منظور انجام پژوهش، 64 کودک در محدوده سنی 13 تا 15 سال در سطوح پیشرفته زبان انگلیسی (سطح RECE و REACH در کانون زبان ایرانیان و سطوح معادل آن در آموزشگاه‌های دیگر) به عنوان گروه مورد (دوزبانه) به همراه 51 کودک غیر زبان‌آموز (تک‌زبانه) در همان رده سنی (13 تا 15 ساله) به عنوان گروه شاهد از منطقه 3 شهر اصفهان انتخاب شدند. کودکان هر دو گروه از آموزشگاه‌های مشابه انتخاب شدند. انتخاب نمونه به‌وسیله روش نمونه‌گیری هدفمند انجام شد. به منظور بررسی و اندازه‌گیری میزان خلاقیت کودکان تک‌زبانه و دوزبانه از پرسش‌نامه خلاقیت تورنس استفاده شد و همچنین از نسخه فارسی کامپیوتری‌شده آزمون استروپ کلاسیک D-KEFS CW (آزمون رنگ‌واژه) برای سنجش و اندازه‌گیری توانایی سوئیچینگ دو گروه کودکان تک‌زبانه و دوزبانه استفاده شد. داده‌های به‌دست‌آمده از پرسش‌نامه خلاقیت تورنس و آزمون استروپ کلاسیک D-KEFS CW، از طریق روش‌های آماری توصیفی و نیز با استفاده از روش آماری تحلیل واریانس چندمتغیره (MANOVA) تجزیه‌وتحلیل شد. تجزیه‌وتحلیل داده‌ها با نسخه 22 نرم‌افزار SPSS انجام شد. یافته‌ها نتایج نشان داد بین کودکان دوزبانه و کودکان تک‌زبانه ازلحاظ متغیر سوئیچینگ تفاوت معناداری وجود دارد (p<0/05). به این صورت که کودکان دوزبانه در تکالیف سوئیچینگ بهتر از کودکان تک‌زبانه عمل کردند. همچنین بین نمره‌های کودکان تک‌زبانه و کودکان دوزبانه در سه مؤلفه خلاقیت که شامل سیالی، انعطاف‌پذیری و بسط است تفاوت معنادار مشاهده شد (P<0/001). به عبارت دیگر کودکان دوزبانه در تکالیف مرتبط با سیالی، انعطاف‌پذیری و بسط بهتر از کودکان تک‌زبانه عمل کردند. از لحاظ مؤلفه ابتکار در دو گروه تفاوتی مشاهده نشد  (0/01 نتیجه‌گیری بر اساس یافته‌های به‌دست‌آمده می‌توان نتیجه گرفت فراگیری زبان انگلیسی در حد پیشرفته و از طریق آموزشگاه‌های زبان می‌تواند به‌طور معناداری توانایی سوئیچینگ کودکان و نمره‌های آن‌ها را در سه مؤلفه خلاقیت (که شامل سیالی، انعطاف‌پذیری و بسط می‌شود) افزایش دهد. از این‌رو باید به نقش فراگیری زبان دوم بر خلاقیت و توانایی سوئیچینگ کودکان توجه شود. به‌طورکلی، عملکرد بهتر کودکان زبان‌آموز را می‌توان به تعویض مداوم زبان در افراد دوزبانه، مکانیسم‌های خاص شناختی استفاده از دو سیستم زبانی، آشنایی کودکان با فرهنگ و آداب‌ورسوم جدید به‌واسطه یادگیری زبان جدید، کسب تجربه‌های جدید که فراگیری زبان تازه به همراه دارد، تمرینات فشرده ارائه‌شده در کلاس‌های آموزش زبان و همچنین جوّ خاص حاکم بر کلاس‌های آموزش زبان نسبت داد.

Title: بررسی میزان همبودی اختلالات رفتاری در کودکان مبتلا به نقص توجه و بیش‌فعالی

http://rehabilitationj.uswr.ac.ir/article-1-1529-fa.html

هدف اختلال نقص توجه و بیش‌فعالی از شایع‌ترین اختلالات روان‌پزشکی در بین کودکان مراجع‌کننده به مراکز درمانی محسوب می‌شود که به بروز مشکلاتی در زندگی اجتماعی و فردی منجر خواهد شد. این پژوهش با هدف بررسی مشکلات رفتاری شایع در کودکان مبتلا به نقص توجه و بیش‌فعالی انجام شده است. روش بررسی این مطالعه توصیفی‌تحلیلی روی 278 کودک مبتلا به اختلال نقص توجه و بیش‌فعالی هفت تا یازده سال انجام شد که از اردیبهشت تا بهمن1391 به مرکز طبی کودکان و کلینیک اعصاب و روان آتیه مراجعه‌کرده بودند. به والدین کودکانی که براساس ملاک‌های چهارمین ویرایش راهنمای تشخیصی و آماری اختلالات روانی (DSM-IV-TR) و با تشخیص روان‌پزشک به اختلال نقص توجه و بیش‌فعالی مبتلا بودند، برای بررسی اختلالات رفتاری همراه پرسش‌نامه علائم مرضی کودکان (CSI-4) ارائه شد. برای توصیف و تحلیل داده‌های به‌دست‌آمده از نسخه نوزدهم نرم‌افزار SPSS استفاده شد. یافته‌ها براساس نتایج به‌دست‌آمده بیشتر مراجعه‌کنندگان از گروه پسران بودند و نقص توجه غالب در دختران و نوع ترکیبی در پسران بیشترین فراوانی را داشت. در هیچ‌یک از اختلالات رابطه معناداری بین جنسیت و نوع اختلال مشاهده نشد. بیشتر آزمودنی‌ها از نظر تعداد اختلال همراه تک‌اختلال بودند. در مقایسه نسبت شیوع اختلالات همراه در سه گروه اختلال نقص توجه و بیش‌فعالی رفتار مقابله‌جویانه (0/0004) و اضطراب منتشر (0/02) بیشترین میزان را داشت. نتیجه‌گیری شیوع اختلالات همراه در سه گروه اختلال نقص توجه و بیش‌فعالی براساس سن و جنس متفاوت بود و بین همبودی این سه نوع اختلال و اختلالات همراه (اختلال رفتار مقابله‌جویانه و اضطراب منتشر) تفاوت معنی‌داری وجود داشت.

Title: تکرارپذیری اندازه‌گیری سطح مقطع عضلات لونگوس‌کولی و لونگوس‌کپیتیس هنگام آزمون کرانیوسرویکال فلکشن توسط اولتراسونوگرافی

http://rehabilitationj.uswr.ac.ir/article-1-1768-fa.html

هدف عضلات گردن بیش از 80 درصد از ثبات مکانیکال ستون فقرات گردنی را تأمین می‌کند. لونگوس‌کولی و لونگوس‌کپیتیس عضلات خم‌کننده عمقی گردن هستند که نقش مهمی در حفظ ثبات گردن ایفا می‌کنند. در بیماران با گردن‌درد مزمن، ضعف و آتروفی این عضلات دیده می‌شود. اندازه‌گیری ابعاد عضله با اولتراسونوگرافی فرصتی فراهم می‌کند تا بتوان آتروفی و هایپرتروفی عضله را به صورت عینی ارزیابی کرد. این مطالعه به منظور بررسی تکرارپذیری اندازه‌گیری سطح مقطع عضلات لونگوس‌کولی و لونگوس‌کپیتیس با اولتراسونوگرافی هنگام انجام آزمون کرانیوسرویکال فلکشن انجام شده است. روش بررسی 10 آزمودنی شامل 5 بیمار با گردن‌درد مزمن غیراختصاصی و 5 فرد سالم به طور داوطلبانه در مطالعه شرکت کردند. تصویربرداری به وسیله اولتراسونوگرافی در محاذات غضروف تیروئید در پنج مرحله با آزمون کرانیوسرویکال فلکشن انجام گرفت. برای بررسی تکرارپذیری درون روز دو تصویر در یک روز با فاصله یک ساعت و برای تکرارپذیری بین روز یک تصویر یک هفته بعد گرفته شد. در هر تصویر سطح مقطع عضلات لونگوس‌کولی و لونگوس‌کپیتیس اندازه‌گیری شد. سپس داده‌های به‌دست‌آمده با استفاده از نرم‌افزار SPSS تحلیل شد. یافته‌ها ICC تکرارپذیری درون روز اندازه‌گیری سطح مقطع عضلات خم‌کننده عمقی گردن با استفاده از اولتراسونوگرافی در افراد سالم 0/91 و در بیماران با گردن‌درد مزمن 0/90 به دست آمد. ICC تکرارپذیری بین روز برای افراد سالم 0/88 و بیماران با گردن‌درد مزمن 0/86 به دست آمد. میانگین خطای اندازه‌گیری حداقل 0/03 و حداکثر 0/05 به دست آمد. نتیجه‌گیری اولتراسونوگرافی در اندازه‌گیری سطح مقطع عضلات لونگوس‌کولی و لونگوس‌کپیتیس هنگام انجام آزمون CCF در افراد سالم و افراد با گردن‌درد مزمن غیراختصاصی روشی پایا و تکرارپذیر است.

Title: تأثیر شش هفته تمرین‌های اصلاحی ساختاری و عضلات تنفسی بر شاخص‌های قلبی و ریوی کودکان پسر 10 تا 12 سال مبتلا به کیفوز

http://rehabilitationj.uswr.ac.ir/article-1-1881-fa.html

هدف ناهنجاری‌های ساختاری بدن می‌تواند بر عملکردهای مختلف بدن تأثیر بگذارد. کیفوزیس یکی از شایع‌ترین ناهنجاری‌های وضعیتی است. عارضه کیفوزیس آثار نامطلوبی بر دستگاه تنفس افراد مبتلا به جا می‌گذارد. اختلال در عملکرد ریوی به‌طور قابل توجهی بر شاخص‌های اسپیرومتری اثرگذار است. پژوهش‌های پیشین تأثیر مثبت تمرین‌های اصلاحی ساختاری را بر عارضه کیفوز نشان می‌دهند. اخیراً تمرین‌های عضلات تنفسی به عنوان یک روش تمرینی در بیماری‌هایی با اختلال عملکرد ریوی در نظر گرفته می‌شود. هدف تحقیق حاضر بررسی تأثیر شش هفته تمرین‌های اصلاحی ساختاری و عضلات تنفسی بر شاخص‌های قلبی و ریوی پسران مبتلا به کیفوز است. روش بررسی جامعه آماری این تحقیق پسران 10 تا 12 سال شهرستان بندرعباس بودند که 34 نفر از پسران مبتلا به کیفوز به صورت دردسترس و با گرفتن رضایت‌نامه از والدینشان انتخاب شدند. زاویه کیفوز آزمودنی‌ها با استفاده از خط‌کش منعطف اندازه‌گیری شد و پس از تأیید ناهنجاری کیفوز وضعیتی مساوی یا بیشتر از 40 درجه، بر اساس درجه کیفوز همتاسازی شدند و به سه گروه تمرین اصلاحی ساختاری (12 نفر)، تمرین عضلات تنفسی (12 نفر) و کنترل (10 نفر) تقسیم شدند. شاخص‌های ریوی آزمودنی‌ها شامل حجم ذخیره دمی(IRV)، حجم ذخیره بازدمی(ERV)، ظرفیت حیاتی اجباری(FVC)، حداکثر تهویه ارادی (MVV) و حجم هوای بازدمی در ثانیه اول (FEV1) با استفاده از اسپیرومتر و حداکثر اکسیژن مصرفی (VO2max) با آزمون 20 متر شاتل ران ، قبل و 24 ساعت بعد از دوره برنامه تمرینی اندازه‌گیری شد. برنامه تمرینی به مدت شش هفته و هر هفته سه جلسه به روش فزاینده اجرا شد. ارزیابی یافته‌ها با استفاده از نسخه 21 نرم‌افزار SPPSS انجام گرفت. پس از تأیید یافته‌ها با آزمون کولموگروف اسمیرونوف، برای مقایسه متغیرهای اندازه‌گیری‌شده در پس‌آزمون سه گروه با کنترل تفاوت‌های احتمالی در پیش‌آزمون، از آزمون تحلیل کوواریانس (ANCOVA) استفاده شد. یافته‌ها نتایج تحقیق نشان داد تمرین‌های عضلات تنفسی موجب افزایش (IRV (P=0/001)، ERV (P=0/001)، FVC (P=0/001 و (MVV (P=0/001 در مقایسه با گروه کنترل و همچنین افزایش شاخص‌های (IRV (P=0/001)، ERV (P=0/001)، FVC (P=0/001 و (MVV (P=0/001 در مقایسه با گروه اصلاحی ساختاری می‌شود، ولی (FEV1 (P=0/126)، VO2max (P=0/49 و درجه کیفوز بین گروه‌ها تفاوت معناداری را نشان نداد (0/38=P). نتیجه‌گیری با توجه به یافته‌های تحقیق حاضر و مشاهده افزایش شاخص‌های قلبی‌ریوی پس از تمرین‌های عضلات تنفسی و آسان‌بودن، نیازنداشتن به وسیله خاصی برای اجرا و مدت زمان کوتاه (شش هفته تمرین)، روش تقویت عضلات تنفسی در بهبود برخی ظرفیت‌ها و حجم‌های ریوی بهتر و کارآمدتر از تمرین‌های اصلاحی ساختاری است. با توجه به اثرات ثابت‌شده تمرین‌های اصلاحی ساختاری در زمینه‌ کاهش زاویه کیفوز در تحقیقات پیشین و عدم تأثیر معنادار آن در تحقیق حاضر، به‌نظر می‌رسد زمان بیشتری (احتمالاً 12 هفته) برای اثربخشی این تمرین‌ها نیاز است. همچنین عدم کنترل عادت‌های غلط حرکتی و وضعیت‌های بدنی مؤثر بر میزان کیفوز در فعالیت‌های روزانه از محدویت‌های تحقیق حاضر است که ارزیابی آن در تحقیقات آینده توصیه می‌شود.

Title: اثربخشی گروه‌درمانی مبتنی بر پذیرش و تعهد بر سازگاری اجتماعی و هراس اجتماعی معلولان جسمی

http://rehabilitationj.uswr.ac.ir/article-1-1942-fa.html

هدف رفتار انسان تابع تمامیت وجود او است. تعامل جسم و روان در رفتار انسان تأثیر می‌گذارد. معلولیت، ناتوانی در انجام تمام یا قسمتی از ضروریات عادی زندگی فردی یا اجتماعی به علت وجود نقص مادرزادی یا عارضی در قوای جسمانی و روانی است. رویکرد توان‌بخشی معلولیت‌ شامل سه دسته ذهنی روانی، حسی و جسمی است. معلولیت جسمی به چهار دسته جسمی حرکتی، احشایی، حسی و سالمندی تقسیم می‌شود. در هر جامعه، با هر وضع فرهنگی و در هر طبقه اجتماعی معلولیت به صورت ناتوانی موقت یا دائم، کلی یا جزئی، ثابت یا متغیر وجود دارد. عوارض ناشی از معلولیت در افراد مختلف با توجه به نگرش آن‌ها متفاوت است. دیدگاه فرد معلول درباره خود و معلولیت و نگرش و برخوردهای خانوادگی و اجتماعی به معلولیت موجب به‌وجودآمدن عوارض گوناگونی در زندگی فرد معلول می‌شود. امروزه ثابت شده است که به‌خطرافتادن سلامت جسمانی موجب بروز واکنش‌های روانی در افراد می‌شود. بسیاری از معلولیت‌های جسمانی با مشکلات روانی در ارتباط است. در واقع، مشکلات روانی در افراد مبتلا به مشکلات جسمانی شیوع بالایی دارد. بیماری‌های مزمن جسمانی شرایط درازمدت و محدودکننده‌ای را برای فرد ایجاد می‌کند که تبعات منفی رفتاری به دنبال دارد. همچنین مشکلات روانی می‌تواند تبعاتی به صورت بیماری‌های شبه‌جسمانی، انواع معلولیت‌ها یا بیماری‌های روان‌تنی به همراه داشته باشد. رشد و گسترش دنیای ماشینی، بالارفتن سن ازدواج و بسیاری مسائل دیگر ممکن است با افزایش معلولیت جسمانی و بیماری‌های روانی همراه باشد. مشکلات روانی در معلولان جسمی با مشکلات جسمانی آن‌ها همراه می‌شود. ازجمله مشکلات روانی که معلولان با آن روبه‌رو هستند، سازگاری اجتماعی پایین و هراس اجتماعی بالا است. هدف از پژوهش حاضر تعیین اثربخشی گروه‌درمانی مبتنی بر پذیرش و تعهد بر سازگاری اجتماعی و هراس اجتماعی معلولان جسمانی بود.  روش بررسی جامعه پژوهش حاضر شامل تمام معلولان حرکتی سازمان بهزیستی شهر قم می‌شد که از میان نمونه‌ای شامل 30 نفر پسر (15 نفر گروه کنترل و 15 نفر گروه آزمایش) با روش نمونه‌گیری تصادفی انتخاب شدند. روش پژوهش نیمه‌آزمایشی بود و شرکت‌کنندگان در مداخله دو ماهه به مدت ۸ جلسه 120 دقیقه‌ای آموزش دیدند. طرح تحقیق از نوع پیش‌آزمون و پس‌آزمون با گروه کنترل بود. داده‌ها با استفاده از پرسش‌نامه‌های سازگاری اجتماعی ویسمن و هراس اجتماعی کانور جمع‌آوری شد. به منظور تحلیل داده‌ها از آزمون تحلیل کوواریانس استفاده شد.  یافته‌ها نتایج نشان داد در افرادی که تحت گروه‌درمانی قرار گرفتند، گروه‌درمانی مبتنی بر پذیرش و تعهد بر سازگاری اجتماعی  مؤثر است (P<0/001). همچنین مداخله گروه‌درمانی مبتنی بر پذیرش و تعهد تأثیر معناداری بر خرده‌مقیاس‌های ترس (P<0/001)، اجتناب (P<0/001)، فیزیولوژی (P<0/001) و نمره هراس اجتماعی (P<0/001) داشت. نتیجه‌گیری گروه‌درمانی مبتنی بر پذیرش و تعهد باعث افزایش سازگاری اجتماعی و کاهش هراس اجتماعی در معلولان جسمی می‌شود.
